# Supplementary figures and images for: UVR8-mediated inhibition of shade avoidance involves HFR1 stabilization in Arabidopsis
Source: PLoS Genet. 2020 May 11;16(5):e1008797. doi: 10.1371/journal.pgen.1008797 (PMC7241853; doi:10.1371/journal.pgen.1008797)

A

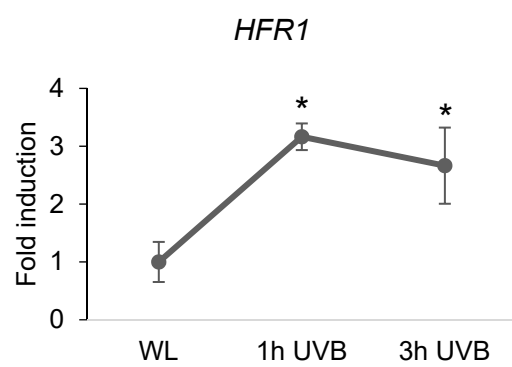

B

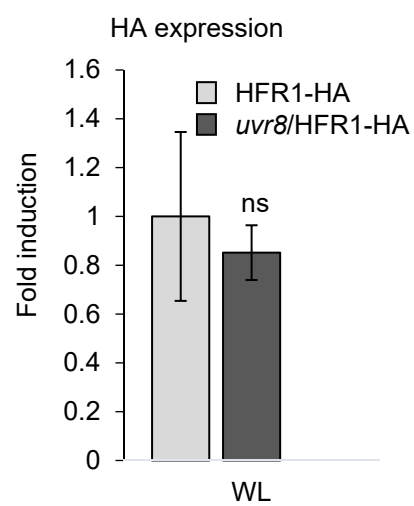

S1 Fig

Supplement: S1 Fig — (A) RT-qPCR analysis of HFR1 expression in 4-day-old wild-type (Col) seedlings exposed to narrowband UV-B for 1 and 3 hours (1-h/3-h UVB) or not (WL). Error bars represent SEM of three independent biological replicates. Asterisks indicate a significant increase in transcript abundance compared to that under WL (*p < 0.05). (B) RT-qPCR analysis of HFR1-3xHA expression in 4-day-old seedlings of Col/ProHFR1:HFR1-3xHA (HFR1-HA) and uvr8-6/ProHFR1:HFR1-3xHA (uvr8/HFR1-HA) grown under white light (WL). Error bars represent SE of three independent biological replicates. (PDF) [file pgen.1008797.s001.pdf]

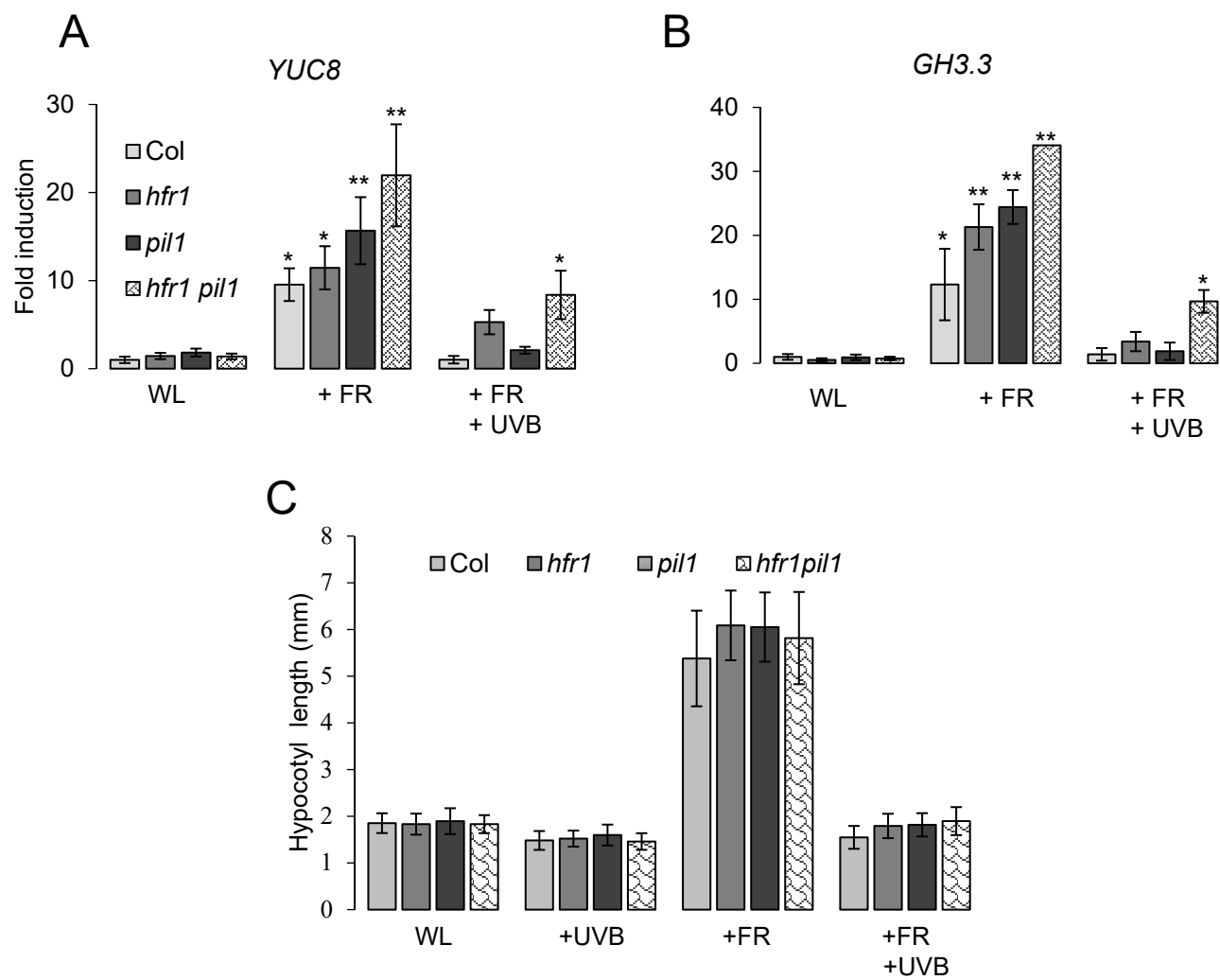

S2 Fig

Supplement: S2 Fig — (A, B) RT-qPCR analysis of (A) YUC8 and (B) GH3.3 expression in 7-day-old wild-type (Col), hfr1-101 (hfr1), pil1-6 (pil1), and hfr1-101 pil1-6 (hfr1pil1) seedlings grown under white light (WL) and exposed to 3-h low R:FR (+FR) or to 3-h low R:FR with supplemental UV-B (+FR +UVB), compared to seedlings maintained under WL as control. Error bars represent SEM of three independent biological replicates. Asterisks indicate a significant difference in transcript abundance compared to that under WL in each genotype (* p < 0.05; ** p < 0.01). (C) Hypocotyl length measurements of Col, hfr1, pil1, and hfr1 pil1 seedlings grown in long-day conditions for 3 days before being transferred to WL, +UVB, +FR, and +FR+UVB for 4 days. Data represent mean length ± SE (n ≥ 40). (PDF) [file pgen.1008797.s002.pdf]

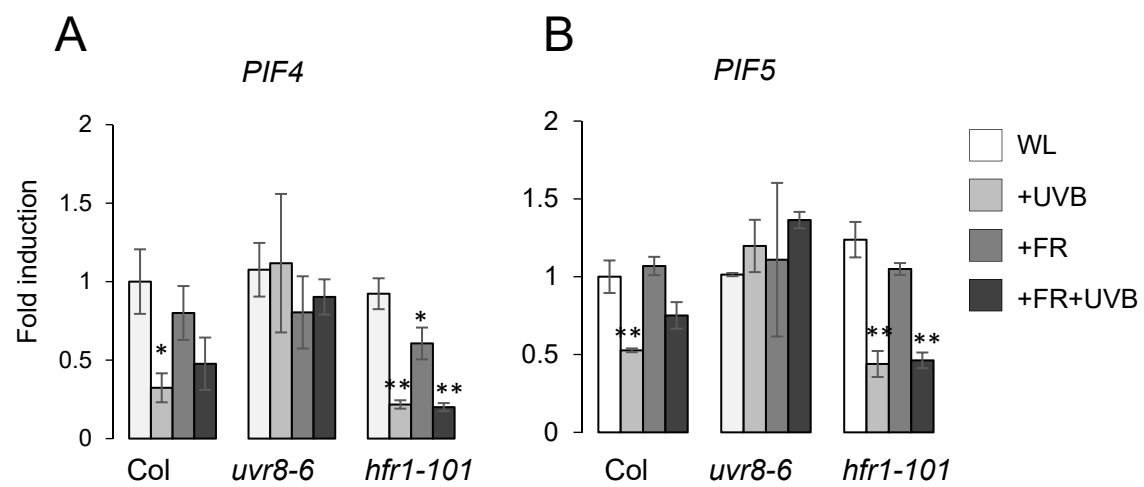

S3 Fig

Supplement: S3 Fig — (A, B) RT-qPCR analysis of (A) PIF4 and (B) PIF5 expression in 7-day-old wild-type (Col), uvr8-6, and hfr1-101 seedlings grown under white light (WL) and exposed to supplemental 3-h UV-B (+UVB), low R:FR (+FR), or low R:FR and UV-B (+FR+UVB) compared to seedlings maintained under WL as control. Error bars represent SEM of three independent biological replicates. Asterisks indicate a significant difference in transcript abundance compared to that of WL in each genotype (* p < 0.05; ** p < 0.01). (PDF) [file pgen.1008797.s003.pdf]

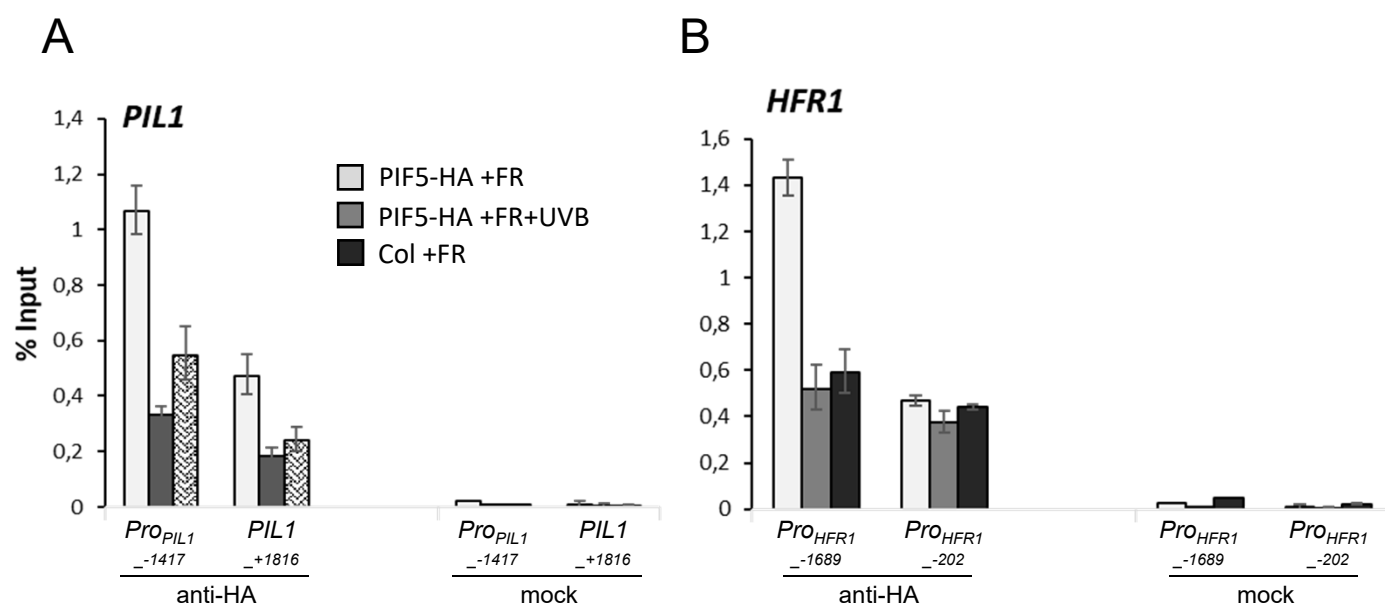

S4 Fig

Supplement: S4 Fig — (A, B) PIF5-HA chromatin association in Col/ProPIF5:PIF5-3xHA (PIF5-HA). Ten-day-old seedlings were grown in long-day conditions under white light and exposed at ZT3 to 3-h low R:FR with or without supplemental narrowband UVB. ChIP-qPCR was performed for (A) PIL1 and (B) HFR1 promoters. The numbers of the analyzed DNA fragments indicate the positions of the 5’ base pair of the amplicon relative to the translation start site (referred to as position +1). Fragments designated as ProPIL1_-1417 and ProHFR1_-1689 contain a G-box, whereas ProPIL1_+1816 and ProHFR1_-202 are devoid of a G-box. ChIP of DNA associated with PIF5-HA is presented as the percentage recovered from the total input DNA (% Input). Data shown are representative of three independent biological replicates. Error bars represent SD of three technical replicates. (PDF) [file pgen.1008797.s004.pdf]

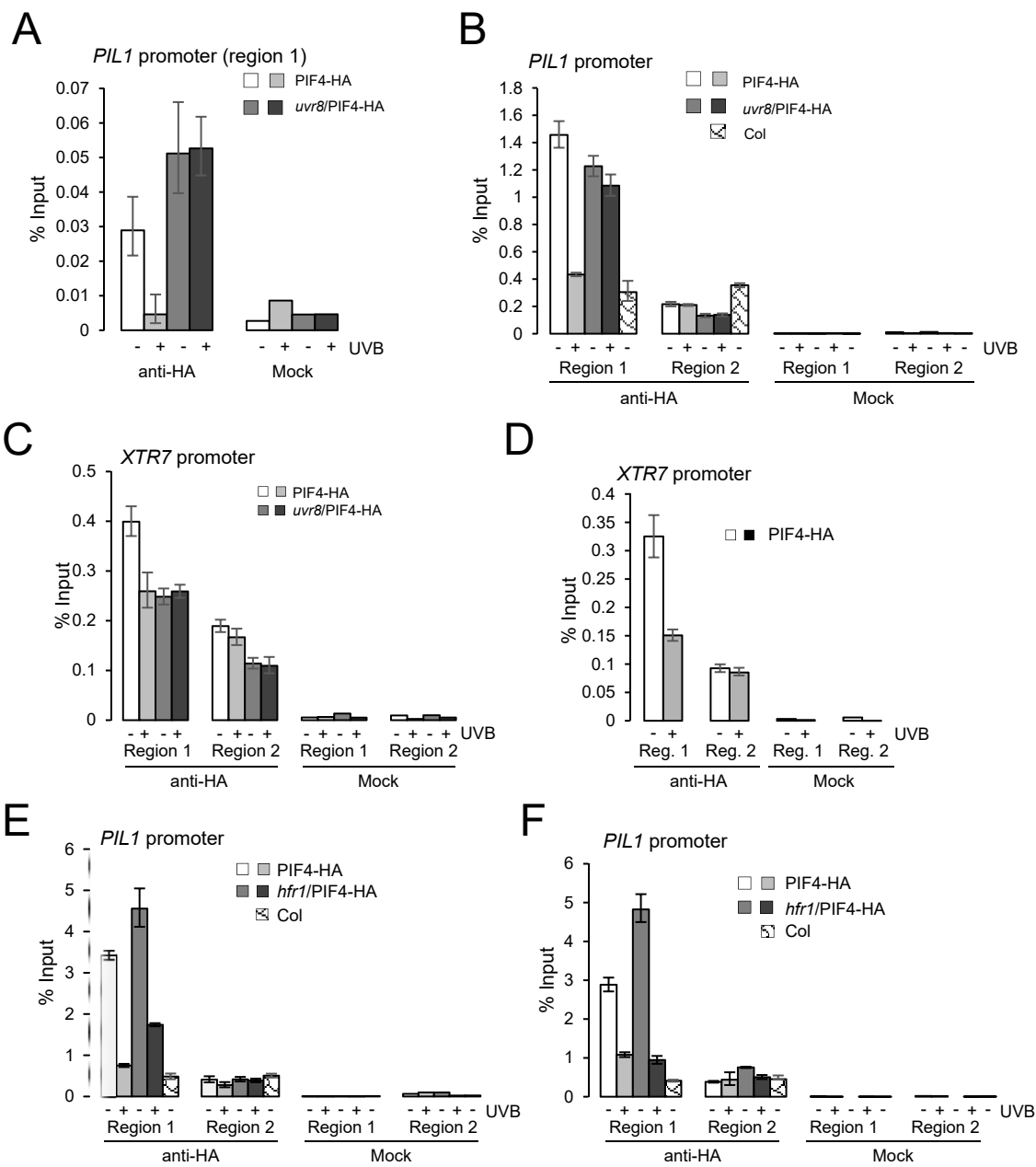

S5 Fig

Supplement: S5 Fig — (A-D) PIF4-HA chromatin association in Col/ProPIF4:PIF4-3xHA (PIF4-HA) and uvr8-6/ProPIF4:PIF4-3xHA (uvr8/PIF4-HA). Ten-day-old seedlings were grown in long-day conditions under white light and exposed to low R:FR with (+) or without (-) 3-h supplemental narrowband UV-B at ZT3. ChIP-qPCR was performed for (A,B) PIL1 (repetitions of data shown in Fig 5B) and (C,D) XTR7 promoters (repetitions of data shown in Fig 5C). (E,F) Chromatin association of PIF4-HA in 10-day-old Col/ProPIF4:PIF4-3xHA (PIF4-HA) and hfr1-101/ProPIF4:PIF4-3xHA (hfr1/PIF4-HA) seedlings grown in long-day conditions under white light and exposed at ZT3 to 3-h low R:FR with (+) or without (-) supplemental UV-B. ChIP-qPCR was performed for the PIL1 promoter (repetitions of data shown in Fig 6C). Error bars represent SD of three technical replicates. (PDF) [file pgen.1008797.s005.pdf]
