# Supplementary material for: UVR8-mediated inhibition of shade avoidance involves HFR1 stabilization in Arabidopsis
Source: PLoS Genet. 2020 May 11;16(5):e1008797. doi: 10.1371/journal.pgen.1008797 (PMC7241853; doi:10.1371/journal.pgen.1008797)
Supplement: Attachment — Submitted filename: Tavridou_PLoSGenet_Response_to_Reviewers_Comments_2020.pdf [file pgen.1008797.s010.pdf]

UNIVERSITÉ DE GENÈVE
FACULTÉ DES SCIENCES
Département de botanique et biologie végétale

Prof. Roman Ulm

Tel.: +41 22 379 36 50
roman.ulm@unige.ch

PLOS Genetics

Geneva, April 6, 2020

Revisions for PGENETICS-D-20-00017

Dear reviewers, dear editors,

Many thanks for your positive and constructive comments on our manuscript entitled "'UVR8-mediated inhibition of shade avoidance involves HFR1 stabilization in Arabidopsis". These comments have helped us to further improve our manuscript, and we are happy to submit a revised version for publication in *PLOS Genetics*.

We have responded to specific reviewer comments and suggestions, as outlined in the detailed point-by-point response to reviewers' comments below. In addition, we provide a marked-up pdf version of the manuscript, in which all changes made during revisions are tracked.

We hope that you approve of the revised manuscript for publication in *PLOS Genetics*. Together with my team members and collaborators, I look very much forward to hearing from you.

Yours sincerely,
Roman Ulm

<u>Point-by-point response to reviewers' comments:</u>

<u>Reviewer #1:</u>

Under FR-rich light, as under a canopy, plants exhibit a shade avoidance response (SAS) leading to enhanced elongation growth. The UV-B receptor UVR8 represses the shade avoidance response under UV-B. This mechanism might lead to a rapid stop in the shade avoidance response when plants grow out of the canopy into the direct sunlight. The molecular mechanisms leading to UV-B-induced suppression of the SAS are poorly understood and currently of great interest to plant biologists.

In this manuscript, the authors demonstrate the important function of the transcription factor HFR1 in UV-B-induced suppression of the SAS. HFR1 is an important repressor of the SAS. The authors show that UV-B enhances the activity of HFR1 by stabilizing the HFR1 protein. They further show that HFR1 is indeed necessary for the UV-B-induced suppression of a subset of SAS marker genes. Indeed, UV-B inhibits binding of PIF4 to the promoter of a subset of shade marker genes and this inhibition is to a large extent dependent on HFR1. Because UV-B induced PIF4 degradation is HFR1-independent, HFR1 activity on PIF4 is independent of the regulation of PIF4 stability.

This manuscript very conclusively uncovers the important role of HFR1 in UV-B signaling under shade conditions. The data therefore very nicely link the activities of UVR8, COP1, HFR1 and PIF4. Their results support the conclusions drawn by the authors. The manuscript is well written.

- *Response:*
We thank reviewer #1 for appreciating our work.

Major comments:

- the molecular phenotype of the hfr1 mutant in +FR+UV-B is very well analyzed and presented. I am wondering about the visible phenotype of an hfr1 mutant in +FR+UV-B in comparison to +FR? I.e. hypocotyl elongation.

- *Response:*

New Figure S2C: we now provide hypocotyl elongation data. Under the tested conditions, there is no visible phenotype of hfr1 and hfr1 pil1 mutants in +FR+UV-B in comparison to +FR.

Minor comments:

- please provide a statistical analysis of the ChIP data.

- Response:
As the % input values vary considerably between fully independent ChIP experiments, we prefer not to combine such data. However, we provide the independent repetitions for the ChIP data in the new supplementary Fig. S5.

- it appears to me that Fig. 6C very nicely demonstrates that UV-B shows a much weaker inhibition of PIF4-binding to the PIL1 promoter in an hfr1 mutant than in the WT – despite no change in PIF4 protein levels in hfr1 vs. WT in +UV-B (Fig. 6A). In my view, this very nicely demonstrates that UV-B – via HFR1 – inhibits the DNA-binding activity of PIF4 – which is consistent with the known activity of HFR1 which heterodimerizes with PIFs. However, the authors do not really make this point in the results section. Is there a reason for that?

- Response:
Such a clear difference between hfr1 and WT has only been observed in two out of three independent repetitions. Lines 198-200 now read: "Although the effect seems reduced in hfr1 mutants, which would be in agreement with the known activity of HFR1 heterodimerizing with PIF4 and PIF5 (11), this has only been clearly observed in two out of three independent repetitions (Fig. 6C and S5E,F).".

Reviewer #2:
The present manuscript by Tavridou et al investigates the molecular details of UVB-mediated inhibition of shade avoidance syndrome (SAS). SAS is induced by low red:far-red (R:FR) light ratios indicating neighbouring plants in close proximity and is characterised by the induction of a well-defined gene set by the PIF4 and PIF5 transcription factors, eventually resulting in accelerated elongation. UVB, perceived by the UVR8 receptor, inhibits this response. The authors showed that UVB promotes the accumulation of the HFR1 transcription factor probably via the UVR8-mediated sequestration of the COP1 E3 ubq ligase, which targets HFR1 for degradation in the dark or in shade conditions. HFR1 forms inactive heterodimers with PIF4 and PIF5 transcription factors that attenuates SAS-related gene expression. They showed that PIL1, another transcription factor implicated in the regulation of SAS acts redundantly with HFR1 in this process. This newly identified molecular mechanism along with the previously described UVB-induced degradation of PIF4 and PIF5 have an important role in the termination of elongation once the plant has overgrown shade.
The experiments are well-planned and executed, providing convincing results. The text is focused, easy to read. The figures are informative and of good quality.

- Response:
We thank reviewer #2 for the positive comments.

I have just a few minor comments and questions to this manuscript. Answers to the questions might be even incorporated in the Discussion.

1. Abstract, line 33: I would say 'PIl1-mediated inhibition of PIF4 and PIF5 function', to indicate that this regulation does not target the expression/level of PIFs.

- Response:
Line 33: Done.

2. I suggest to quantitate signals on all Western-blots (not only the one in Fig.6). Statistical analysis of these data would provide a robust support for the key statements/findings.

- Response:
Fig. 4: We provide now the quantification of the western blots. It is of note that the finding of PIF4 and PIF5 degradation is in agreement with similar findings published by Hayes et al. 2014 and 2017, as well as Sharma et al. 2019; as we cite e.g. lines 91/92: "Molecularly, UVR8 activity was associated with the degradation of PIF4 and PIF5 (27, 28, 32, 36), the key inducers of SAS (3, 8, 37).".

3. Fig.S4 seems to be not finalized yet: „devoid G" should be changed to „devoid G-box" (and explain this in the legends), and indicate HA-derived or mock immunoprecipitation in panel A as well.

- Response:
Figure S4 and lines 520-523: We have corrected the labelling of the figure, as well as its figure legend.

4. Fig.2B demonstrates that a significant number of genes are induced by FR in the uvr8 mutant, but not in Col. How could the authors explain this if UVR8 was told to have function in UVB only?

- Response:
In many cases this is due to the FC > 2 & adjusted pvalue < 0.05 criteria (see Table S2, "uvr8_FR/WL_up"). To determine if there are indeed a few genes that are truly and reliably FR induced in uvr8 mutants but not in wild type or hfr1 needs careful verification by qPCR. We consider such an in-depth analysis out of scope of the present manuscript.

5. Fig.2 deals with shade-induced genes. Did the authors identify shade-repressed genes in their RNA-seq assay? If yes, how was the repression affected by the UVB treatment?

- Response:
PIF5 is mostly involved in activating shade-induced genes (direct effect) based on comparisons of ChIP-Seq and gene expression data (e.g. Hornitschek et al., 2012). The publication by Leivar et al., 2012 is also consistent with PIFs primarily acting as activators of gene expression (direct effects). However, it cannot be ruled out that during shade-avoidance PIF4 and PIF5 also directly repress genes; but this remains an open question despite quite extensive work on PIF4 and PIF5 in shade avoidance responses. To address direct effects of PIF4 and PIF5 in repressing genes in the shade avoidance response is out of scope of this work, and we thus concentrate on the effect of UVR8 signaling on the known activity of PIF4 and PIF5 as activators of gene expression.
